# Smith-Lemli-Opitz Syndrome (SLOS)—Case Description and the Impact of Therapeutic Interventions on Psychomotor Development

**DOI:** 10.3390/jcm14238569

**Published:** 2025-12-03

**Authors:** Natalia Kozera, Robert Śmigiel, Anna Rozensztrauch

**Affiliations:** 1 Department of Pediatrics and Coordinated Child Care, Wroclaw Medical University, 50-367 Wroclaw, Poland; nataliakozera@icloud.com; 2 Department of Pediatrics, Endocrinology, Diabetology and Metabolic Diseases, Wroclaw Medical University, 51-618 Wroclaw, Poland; robert.smigie@umw.edu.pl

**Keywords:** Smith-Lemli-Opitz syndrome, SLOS, DHCR7, interdisciplinary therapy, metabolic disease, rare diseases, cholesterol supplementation, psychomotor skills, psychomotor development

## Abstract

**Background/Objectives**: Smith–Lemli–Opitz syndrome (SLOS) is a genetic metabolic disorder characterized by impaired cholesterol synthesis and a wide range of developmental anomalies. This article presents a case of a girl with SLOS, diagnosed with two pathogenic variants of the DHCR7 gene. The objective is to evaluate the impact of early, multidisciplinary therapeutic interventions on the patient’s development. **Methods**: Following diagnosis, a comprehensive metabolic therapy was initiated, including cholesterol and cholic acid supplementation. An interdisciplinary therapeutic approach was employed, involving physical therapy, speech therapy, and sensory integration, aimed at addressing various developmental challenges faced by the patient. **Results**: The therapy led to gradual improvements in the patient’s psychomotor development, although the cholesterol levels were only partially improved and the accumulation of sterol precursors (7-DHC and 8-DHC) persisted. The coordinated care model facilitated better outcomes compared to less integrated efforts. **Conclusions**: The results highlight the importance of early diagnosis and integrated care in optimizing developmental outcomes for children with SLOS. A multidisciplinary approach is essential for addressing the complexities of the syndrome and promoting overall development.

## 1. Introduction

Smith–Lemli–Opitz syndrome (SLOS) was first described in 1964 by pediatrician David Smith, physician Luc Lemli, and geneticist John Opitz. They observed three boys with microcephaly who shared facial dysmorphic features, anomalies of external genitalia, severely impaired psychomotor development, and feeding difficulties. An alternative name for the syndrome, RSH syndrome, is derived from the initials of the surnames of these patients [1,2,3].

The estimated prevalence of SLOS in live births ranges from 1 in 15,000 to 1 in 60,000 in European populations. However, studies conducted in Poland suggest a higher frequency. Screening for carrier status of DHCR7, the gene which is responsible for SLOS, indicated a carrier frequency of approximately 2.4% in the Polish population, corresponding to an estimated incidence of 1 in 2300 to 1 in 3937 live births. Due to a variable clinical presentation, especially in milder forms of the syndrome, and the high fetal and neonatal lethality, SLOS is frequently underdiagnosed. The wide spectrum of phenotypes underscores the importance of molecular testing for accurate diagnosis and early intervention [4,5,6].

SLOS is a genetically determined metabolic disorder inherited in an autosomal recessive pattern. The molecular defect underlying SLOS results from pathogenic variants in the DHCR7 gene, which encodes 7-dehydrocholesterol reductase (7-DHCR)—an enzyme responsible for the final conversion step of 7-dehydrocholesterol into cholesterol during biosynthesis. Mutations of this gene reduce or abolish enzyme activity, consequently impairing cholesterol production [7]. The DHCR7 gene, mapped to chromosome 11, is composed of 12 exons [8] and eight introns [9]. In affected individuals, most pathogenic variants are missense or nonsense mutations, leading to partial or complete loss of enzymatic function. Notably, over 90% of identified mutations cluster within exons 4, 6, and 9 of the gene [10]. Research conducted by G.S. Tint in 1993 revealed abnormalities in cholesterol biosynthesis, which is the underlying cause of the clinical symptoms of SLOS [11]. Children with RSH syndrome are unable to produce cholesterol, a crucial dietary component, which significantly affects their fetal development, leading to numerous congenital anomalies and impacting brain development. Due to impaired cholesterol synthesis, abnormal sterols accumulate, which results in structural and functional abnormalities in various organs. Numerous studies have shown that cholesterol is essential for the body’s functioning. It is derived not only from food but also synthesized by the liver, playing a vital role in many biochemical processes, including (the synthesis of vitamin D3 [12]. Furthermore, as a metabolite essential for the proper development of all eukaryotic organisms, cholesterol is a fundamental component of cell membranes and myelin, and a primary substrate for the synthesis of other steroids and sterols [13]. In SLOS, abnormal levels of cholesterol are linked to a deficiency of the 7-dehydrocholesterol reductase enzyme (DHCR7), which reduces the Δ7 double bond in 7-dehydrocholesterol (7-DHC), leading to the formation of desmosterol and cholesterol. Disruption of this process results in low cholesterol levels and an elevated concentration of 7-DHC and its isomer, 8-dehydrocholesterol (8-DHC) [14]. The gene which encodes Δ7-dehydrocholesterol reductase is DHCR7, located on chromosome 11, and it is the only known gene whose variants are responsible for the occurrence of SLOS [15]. More than 218 mutations in the DHCR7 gene have been identified, and it is believed that many more may arise because of deletions, mutations, and insertions.

A diagnosis of SLOS based on clinical symptoms should be confirmed through biochemical and/or molecular analysis. This involves blood tests that measure cholesterol levels, as well as concentrations of 7-DHC and 8-DHC in blood serum or skin fibroblasts using the gas chromatography coupled with mass spectrometry (GC-MS technique). In some cases, DHCR7 gene sequencing is performed to confirm the diagnosis, especially when the patient’s clinical presentation suggests SLOS but cholesterol levels fall within the normal range. It is also possible to detect the syndrome through prenatal diagnostics using urine testing. In 1999, it was discovered that the urine of mothers carrying a child with SLOS contains unusual steroid metabolites, which allows the disease to be detected as early as the 9th or 10th week of pregnancy [16]. 

Another method of prenatal diagnosis also exists; however, it uses more invasive techniques, such as chorionic villus sampling or amniocentesis. These procedures allow for assessing cholesterol levels, as well as 7-DHC and 8-DHC concentrations in amniotic fluid. Additionally, cultured cells can be used to identify the specific type of mutation of the DHCR7 gene [17].

In Poland, a biochemical diagnosis of SLOS is conducted by the Laboratory of Steroid Hormones and Metabolic Disorders [Pracownia Hormonów Steroidowych i Zaburzeń Metabolizmu Instytutu] at the “Pomnik—Centrum Zdrowia Dziecka” Institute (IPCZD).

Since these tests are highly specific to the syndrome and parents are often unaware of the possible defects, they are not part of the standard set of prenatal screenings. Therefore, the initial diagnosis of the disease usually occurs after birth, based on the patient’s characteristic clinical presentation.

The clinical overview of SLOS includes a wide range of defects and disorders, which include:

- Microcephaly
- Major developmental abnormalities of the central nervous system and/or cortical structure disorders
- Cataracts or microphthalmia
- Cleft uvula/submucous cleft palate/cleft of the hard palate/cleft lip
- Large vessel defects or single-chambered heart
- Hypoplasia or reduced number of lung lobes
- Pyloric stenosis
- Hirschsprung’s disease
- Isolated structural abnormalities or progressive liver dysfunction
- Renal agenesis or clinically significant polycystic kidney disease
- Hypospadias
- Ambiguous or female external genitalia in individuals with 46, XY karyotype
- Underdevelopment of external genitalia in individuals with 46, XX karyotype
- Y-shaped syndactyly of the second and third toes; other syndactylies, polydactylies, and foot deformities [2].

Treatment of SLOS is symptomatic and depends on specific impairments present in each patient. Due to the wide range of possible symptoms, collaboration amongst multiple specialists is essential, including cardiologists, dentists, neurologists, gastroenterologists, and dietitians. Due to autism spectrum disorders and behavioral issues frequently occurring in SLOS, psychological therapy or psychiatric care may also be necessary. However, treatment should primarily focus on diet, which involves appropriate cholesterol supplementation in both natural and synthetic forms.

The aim of this paper is to present a case of a child with Smith–Lemli–Opitz syndrome (SLOS) and to evaluate the impact of early, multidisciplinary therapeutic interventions. The study highlights the role of cholesterol supplementation, dietary management, physiotherapy, speech and language therapy, sensory integration, and educational support in promoting psychomotor development, based on clinical observations and standardized assessments.

## 2. Materials and Methods

During the patient’s first year of life, psychomotor development was systematically monitored each month. The primary diagnostic instruments included developmental charts designed by Professor Jagoda Cieszyńska and Marta Korendo, facilitating a structured evaluation of the child’s developmental trajectory. These tools provided detailed insights into strengths, acquired competencies, and persisting deficits, particularly in the domain of cognitive functioning.

A baseline speech and language assessment was performed, complemented by a preliminary screening of sensory integration processing. In subsequent years, a developmental follow-up was conducted using the Psychomotor Development Assessment Sheets (KORP), a standardized diagnostic tool for evaluating psychomotor abilities in children.

The individualized therapeutic program was multimodal and interdisciplinary in nature. Physiotherapeutic interventions included the Vojta method and Neurodevelopmental Treatment (NDT, Bobath concept), both targeting global motor function and neuromuscular coordination. Neurological speech therapy was implemented with a dual focus: managing oropharyngeal dysphagia and facilitating oral motor skills. Therapeutic strategies were derived from the Rodolfo Castillo Morales concept, combined with desensitization of the tactile system through sensory integration therapy, and complemented by feeding therapy in accordance with the Human Touch approach.

Adjunctive interventions included Shantala massage, introduced in infancy and carried out regularly by family members, and elastic therapeutic taping performed in line with Esther de Ru’s methodology. Cognitive stimulation was supported through the method of work centers, which was adapted to the developmental profile of a young child. In parallel, the verbal-tonal method was used to enhance auditory and speech perception. After the first year of life, augmentative and alternative communications (AAC) were introduced using Makaton signs, providing additional communicative support. From the second year onward, core therapeutic strategies were further supplemented by regular transcranial direct current stimulation (tDCS) sessions, aiming to modulate cortical excitability and promote neuroplasticity. tDCS sessions lasted 30 min over a 10-day period and were conducted at three-month intervals. The first goal of stimulation was to improve concentration, attention, and memory, reduce psychomotor agitation, and enhance cognitive stimulation and intellectual development. For this purpose, the electrodes were positioned according to a protocol that placed the anode in the Left Cerebral hemisphere and the cathode in the right frontal region of the brain. Subsequent stimulations were aimed at additional stimulation of Broca’s speech center. For this purpose, the cathode was still placed on the right anterior part, and the anode on the posterior part of the inferior frontal gyrus in the left hemisphere, overlapping with Brodmann areas 45 and 44, near the speech motor area. Constant current used was 1 mA.

### Multidisciplinary Care

Managing Smith–Lemli–Opitz syndrome (SLOS) requires a comprehensive, multidisciplinary approach to address the complexity of clinical manifestations and optimize long-term outcomes. A coordinated model of care—integrating medical specialists, rehabilitation professionals, and family education—is essential for improving quality of life, promoting psychomotor development, and fostering greater independence in affected children.

Early and accurate diagnosis, ideally made by a clinical geneticist or a specialist in inborn errors of metabolism, is fundamental. Timely recognition not only broadens the therapeutic window but also enables appropriate coordination of care, systematic monitoring, and targeted family counseling.

A hallmark of SLOS in infancy is feeding dysfunction, characterized by impaired sucking and swallowing reflexes, gastroesophageal reflux, and an exaggerated gag reflex. These complications frequently necessitate the use of alternative feeding methods, such as nasogastric tubes or percutaneous endoscopic gastrostomy. Close collaboration with a gastroenterologist and clinical dietitian is crucial to ensure adequate caloric intake, particularly given the frequent occurrence of failure to thrive and slow weight gain during the first year of life. In many cases, high-calorie nutritional supplements or specialized diets are required to meet developmental energy demands.

Feeding difficulties and reflux may contribute to recurrent otitis media, which can, in turn, cause conductive hearing loss. Consequently, regular otolaryngology (ENT) evaluations are necessary. Dental and maxillofacial care also form a key component of therapy, particularly in children presenting with craniofacial anomalies such as micrognathia, retrognathia, cleft palate, or malocclusion.

Given the frequent co-occurrence of ophthalmological and cardiovascular abnormalities in SLOS—including myopia, strabismus, congenital heart defects, and valvular dysfunction—routine follow-up by ophthalmologists and cardiologists is mandatory.

Ultimately, an effective care model for SLOS relies on structured, interdisciplinary collaboration involving geneticists, metabolic specialists, neurologists, cardiologists, gastroenterologists, otolaryngologists, ophthalmologists, dentists, dietitians, physiotherapists, occupational therapists, and speech-language pathologists. Such a holistic approach ensures that developmental, nutritional, sensory, and systemic needs are addressed in a coordinated manner, supporting both the child’s functional abilities and the family’s capacity to provide ongoing care [12,13,14,15,16,17].

## 3. Results

### 3.1. Patients’ Characteristics

The proband was a female infant born at 39 weeks of gestation via cesarean section. The pregnancy was clinically uneventful, and a routine prenatal ultrasound did not reveal major structural abnormalities. Fetal biometric measurements of limb length were at the lower threshold of normal but not considered pathological.

At birth, the patient weighed 3090 g with a head circumference of 34 cm. Apgar scores were 7/8/8/8 at 1, 3, 5, and 10 min, respectively. Initial physical examination demonstrated generalized hypotonia and microcephaly, in addition to multiple congenital anomalies:

- Craniofacial dysmorphisms: slanted palpebral fissures, broad nasal bridge, low-set ears, and a short neck with a cutaneous fold.
- Extremity anomalies: syndactyly of the 2nd and 3rd toes bilaterally, short limbs, and a transverse palmar crease on both hands.
- Other malformations: hemangiomas on the forehead, eyelids, and nose; submucous cleft palate.

Newborn hearing screening indicated abnormalities in the right ear, warranting further audiological assessment. Cardiac evaluation revealed mild hypokinesia of the myocardium and tricuspid valve insufficiency, not requiring immediate cardiological intervention. Because of multiple congenital anomalies, the staff transferred the neonate at 7 h of life from the private Medfemina Obstetrics and Gynecology Clinic to the 1st Department of Gynecology and Obstetrics, Wrocław, for extended diagnostic work-up.

**Figure 1:**
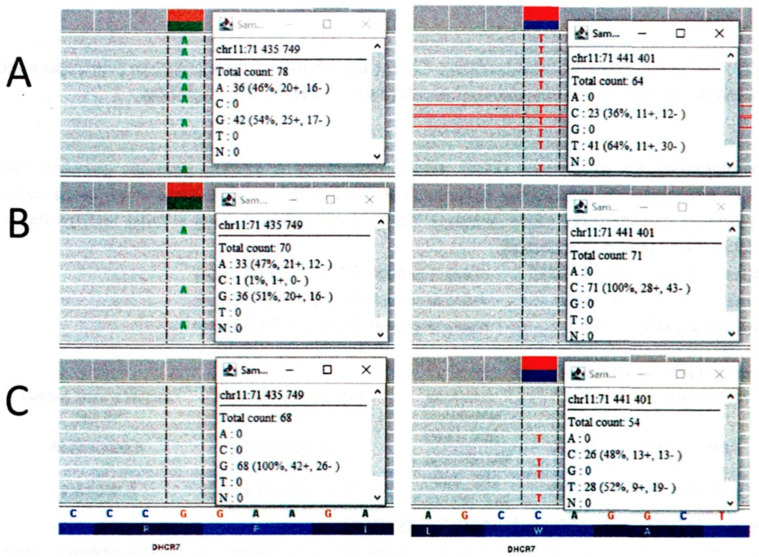
Graphical presentation of the WES result in the DHCR7 gene. (**A**)—proband; (**B**)—mother; (**C**)—father.

Comprehensive genetic testing was subsequently undertaken (Figure 1). The waiting time for WES (Whole Exome Sequencing) and WES trio test results varies depending on the center performing the test. The WES trio test was performed at the Medical University of Warsaw, test was performed after consultation with a geneticist. The sample was collected about two months after the baby’s birth, and the results were received three months later. Whole exome sequencing confirmed the clinical suspicion of Smith–Lemli–Opitz syndrome (SLOS). The patient waited five months for the diagnosis to be confirmed by genetic testing, which is the standard waiting period. The examination confirmed that the patient was found to carry two pathogenic variants in the DHCR7 gene, consistent with a compound heterozygous trans configuration:

- c.1054C > T (classified as pathogenic; causative of SLOS in an autosomal recessive inheritance pattern).
- c.452G > A (classified as pathogenic/probably pathogenic according to ClinVar, v. 13 July 2021).

Molecular findings provide definitive evidence for the diagnosis of autosomal recessive SLOS, correlating with the patient’s phenotypic manifestations.

### 3.2. Metabolic Supplementation and Biochemical Monitoring

Following diagnosis, a comprehensive metabolic and nutritional supplementation was initiated, including vitamin D_3_, B-group vitamins, probiotics to support intestinal microbiota function, as well as oral synthetic cholesterol with 20 mg of it per kilogram of body weight, divided into three doses during the day and cholic acid. This therapeutic strategy aimed to correct cholesterol deficiency, reduce toxic precursor accumulation, and improve overall metabolic homeostasis.

### 3.3. Cholesterol and Sterol Precursor Dynamics

Serial biochemical monitoring demonstrated progressive elevation of plasma cholesterol levels following supplementation (Table 1). However, despite this improvement, 7-dehydrocholesterol (7-DHC) and 8-dehydrocholesterol (8-DHC) levels remained markedly elevated and even showed an increasing trend over time. This biochemical pattern illustrates the partial responsiveness of cholesterol metabolism to supplementation in SLOS and underscores the persistence of DHCR7 enzymatic block.

Although cholesterol rose from 17.4 mg/dL to >100 mg/dL, levels remained significantly below the pediatric reference range (typically >120 mg/dL). Concomitant elevation of 7-DHC and 8-DHC highlights the continued biochemical instability characteristic of SLOS. Importantly, these sterol precursors are prone to auto-oxidation, generating oxysterols that exert cytotoxic and neurotoxic effects, contributing to developmental delay and organ dysfunction. Lipid Fractionation and Related Parameters. Further biochemical profiling included lipid fractions, vitamin D_3_ status, and triglycerides (Table 2).

Analysis of lipid fractions demonstrated persistently low HDL cholesterol (25–34 mg/dL), a finding associated with impaired reverse cholesterol transport and heightened cardiovascular risk. LDL cholesterol remained modest (43–68 mg/dL), while triglycerides were within normal pediatric limits. Notably, vitamin D3 levels were consistently elevated, reflecting effective supplementation.

Taken together, these findings highlight the paradoxical metabolic phenotype of SLOS: despite supplementation, cholesterol levels increase but remain suboptimal, while abnormal sterol intermediates accumulate. This metabolic disequilibrium contributes to multisystem pathology, particularly in the central nervous system, where cholesterol is indispensable for myelination, synapse formation, and neurotransmitter function [18].

### 3.4. Clinical Course and Interventions

Before her first birthday, the patient underwent two surgical procedures: repair of the submucous cleft palate and placement of a percutaneous endoscopic gastrostomy (PEG). The latter was necessitated by profound feeding difficulties, including absent sucking and swallowing reflexes and a persistent exaggerated gag reflex. Initially, feeding consisted of breast milk and formula via bottle and tube, but exclusive tube feeding became necessary before three months of age. The girl exhibited feeding difficulties from the first days of her life. Sucking and swallowing difficulties, present from the very beginning, necessitated the introduction of nasogastric tube feeding, while lack of progress led to the insertion of a gastrostomy tube. Cleft soft palate surgery gave the patient better swallowing capabilities and had a positive impact on reducing the number of regurgitation episodes after feeding. Feeding therapy, based on proper swallowing, biting, gnawing, and chewing, was an integral part of the therapy. It included manual work on the oral area, including massages to desensitize the tactile area and exercises engaging the muscles of the lips, cheeks, tongue, soft palate, and larynx. Currently, the child no longer requires PEG feeding and feeds primarily orally, with occasional alternative feeding, most often during infections. Progress in nutrition is one of the most important things that the patient has achieved. Beginnings of oral feeding included minimal amounts of 5–10 g of thick fluid; currently, the child eats meals of all consistencies with minimal use of gastrostomy during infections or to administer larger amounts of fluids (100 mL of fluids per kilogram of body weight).

Neurological examination revealed generalized hypotonia, absent primitive reflexes (sucking, rooting, swallowing, crawling, Babkin), and markedly diminished protective and postural reflexes (Moro, Galant, auriculopalpebral, oculopalpebral, crossed extensor).

### 3.5. Early Neurodevelopmental Therapy

Therapeutic stimulation began within the neonatal period, utilizing the Castillo Morales concept (RCM) and Bobath NDT. Initial interventions focused on improving muscle tone and feeding. Over the first year, therapy expanded to cognitive training, speech–language stimulation, and oral motor rehabilitation.

A 2-month-and-2-week-old girl. Initial examination revealed hypotonia, lack of postural control, and absent sucking, searching, biting, and chin reflexes. Good eye contact, limited ability to follow, and an exaggerated gag reflex (already present in the shoulder area). She was tube-fed and PEG-fed using Nutricia Infantrini Peptisorb and breast milk. Speech therapy revealed a retrognathic jaw, abnormal resting position of the lips, tongue, and jaw, and reflux. Another examination at 4 months of age. After oral stimulation and Shantala massage, as well as regular physical therapy, the following findings were observed: acceptance of orofacial stimulation, no grasping, lack of control when lying on the stomach, no midline alignment of the hands, and an extensor pattern in the elbows. No vocalizations, frequent crying (most likely caused by reflux discomfort), retrognathia, improved resting position of the lips and tongue, social laughter, and recognition of people around him. A history of frequent constipation and defecation difficulties. Next progress assessment at 6 months of age. Active foot grasp reflex, Babinski’s reflex, gag reflex only when touched inside the mouth, oral stimulation implemented, and food given approximately 5–10 mL orally, first attempts at biting, no active support on the abdomen. At 9 months of age, the child demonstrated a dominant extension pattern in the elbows, better control of weak postural muscles, improved support, increased activation in sitting, a decreased gag reflex, and acceptance of pureed food. Cognitive assessment revealed a lack of pointing, an inability to turn pages in a book, and no babbling, only guttural sounds. Assessment at 12 months showed normal resting position of lips and tongue, eye contact, attempts at babbling, understanding simple “take” commands, turning pages in a book, pointing with eyes supported by a finger when prompted, initial attempts at pointing to body contours, attempts at imitation, and introduction of alternative communication gestures. Approximately 50 mL of food was administered orally, reflux was present, and the Babinski reflex was present. The girl was able to stand upright but had not fully mastered sitting upright; she still requires support and reassurance.

By 12 months, the patient had achieved milestones including head control, rolling, forearm support, crawling, quadruped posture, object manipulation, and early sitting balance. Social contact and nonverbal communication skills were developed appropriately, though expressive language lagged. At 14 months of age, she attempted to sit independently, had improved body image awareness, better eye control, showed numerous oral self-stimulations, attempted biting, mastered drinking from an open cup, and was able to properly scoop food from a spoon. She orally administered 70 mL of food at one meal. At 17 months of age, the patient demonstrated body image awareness, pointing, understanding simple commands, babbled, did not build a tower, sought a person when asked, played peek-a-boo, responded to her name, and sat steadily. When she was 19 months, the patient attempted to stand, crawled, and had improved hand-eye coordination, but was clumsy with movement and was able to stand upright. Speech therapy revealed biting, but no proper biting. She drank from a cup and water bottle, did not drink through a straw, babbled, and orally consumed 70 to 120 mL of food, which contained pieces and lumps. She has been introduced to playing with objects as intended (cooking in pots, cutting with a knife, painting with crayons, etc.), connecting two blocks, building a tower, and learning basic colors (yellow, red, green). At 24 months of age, the patient is sitting independently, crawling, standing, and walking sideways. She is learning to walk from island to island and stand. Postural control is improved, but coordination skills are still weaker. She is trying to eat and drink from a cup independently, and her eye-hand coordination is improving. She points to body parts, imitates actions, recognizes colors, plays with toys as intended, and tries to throw a ball. Emotionally, her cognitive development is normal, with a cognitive delay of +/− 6 months, and her motor skills are estimated at 11–13 months. She has mastered the consistency of puree, mastered drinking of all consistencies, bites, and is increasingly eating solid foods, and has no gagging reflex or gagging. She doesn’t catch a ball or build a tower of blocks. Compared to her peers, her vocabulary was poor. Two-year-olds typically have a vocabulary of 50 to 200 words. Patient’s speech comprehension included simple commands, all everyday objects, and simple actions. Her speech expression included onomatopoeic expressions and sign language gestures, including “give, eat, finish, more, play”. A combination of gestures and onomatopoeia, a few simple words still amounted to less than 50 words.

In the following months of therapy, the focus is primarily on improving the cognitive and motor skills the child has mastered and expanding his vocabulary. TDCS stimulations were introduced.

### 3.6. Ongoing Development

Progress continued across domains, though with persistent motor coordination deficits and speech delay. Improvements in feeding therapy enabled more physiological oral functions: correct jaw–tongue resting position, control of salivation, nasal breathing, and improved swallowing. No features suggestive of autism spectrum disorder were observed. Ophthalmological assessment confirmed myopia and astigmatism, while hearing remained within normal limits.

At 2.5 years, psychomotor evaluation placed development at a 13-month level. By 3.5 years, reassessment indicated a developmental level of 24 months, demonstrating gradual but measurable progress. By age 4, the patient could walk independently with a wide-based gait, use AAC gestures, comprehend language effectively, and produce single words and sound imitations. Adaptive skills included toilet training, participation in preschool activities, and independent feeding with decreasing assistance, although gastrostomy feeding and a high-fat, high-cholesterol diet remained necessary. For the special nutrition provided by the PEG, the child’s diet included products administered orally. The blended meals were rich in foods such as egg yolk, avocado, butter, dairy products, and oils, including coconut oil, olive oil, and goose fat. Each of the meals offered for oral consumption during the day contained from 10 g to 50 g of the selected product.

## 4. Discussion

The patient’s current abilities are the result of multidisciplinary collaboration. The creation of a coordinated medical and therapeutic team overseeing skill acquisition has brought remarkable results and given the child the opportunity to function much better than initially expected in the early months. Limiting therapeutic interventions or delaying consultations with any of the specialists could have led to later achievement of key developmental milestones. Currently, thanks to a wide range of diagnostic and therapeutic support, the child can attend a facility with intellectually typical peers and, moreover, significantly exceeds the psychomotor abilities of many patients diagnosed with SLOS, who often do not demonstrate similar skills at the same age.

Psychomotor developmental delays and autistic traits are common symptoms of SLOS. A late diagnosis and significant developmental delay can lead to further consequences, such as aggressive and self-injurious behaviors and intellectual disability. As noted in research by Audrey Thurm, due to the low functioning level of patients with SLOS, the results of standard autism assessments may be artificially elevated [19]. This means that if patients were to receive diagnoses earlier, along with appropriately tailored treatment and therapeutic plans, their cognitive and motor functioning levels could be significantly different. Autistic traits themselves are not necessarily linked to cholesterol levels or supplementation with exogenous cholesterol. In his research, Peter A. Kaub emphasizes that blood cholesterol levels were not correlated with the severity of ASD symptoms or adaptive behavior in SLOS cases, even after oral cholesterol supplementation. No correlation was found between baseline levels of 7-DHC and 8-DHC and the presence or severity of ASD symptoms [20]. Regardless of whether cholesterol supplementation can significantly impact a patient’s development, without a diagnosis confirmed by genetic testing and without appropriate developmental stimulation, a child will not be able to independently realize their intellectual potential.

The nature of the symptoms observed in patients with SLOS clearly indicates the necessity for prompt therapeutic and medical intervention. As reported by Nowaczyk et al. [21], 90% of patients exhibit impaired or restricted growth, 50% have congenital heart defects, 40–50% are born with a cleft palate, and a similar percentage presents hypotonia. Additionally, 25% have kidney anomalies. All these symptoms may require medical intervention, including surgery. Furthermore, the author notes that most patients require feeding support, alternative methods of nutrition, and suffer from constipation and sleep disorders, which also demand pharmacological management. Deficits in oral-motor coordination, hypotonia, food intolerances, reflux, and Hirschsprung’s disease, commonly observed from the earliest days of life, necessitate consultations with both gastroenterologists and speech therapists. Collaboration between these two specialties is essential to ensure safe and effective nutrition for the patient.

Other authors, such as de Clemente et al. [22], highlight cognitive function deficits and behavioral issues in patients. The range of intellectual disability in individuals with SLOS varies from borderline impairment to severe forms of intellectual disability. Moreover, patients often experience sleep disorders and behavioral disturbances with aggressive and self-injurious tendencies [23,24,25].

Compared with previously reported SLOS cases, such as those described by Nowaczyk et al., the developmental progress in our patient appears relatively rapid (e.g., 11 months of developmental gain over 1 year). Nowaczyk et al. [21] highlighted the heterogeneity of SLOS and noted that response to interventions such as cholesterol supplementation can vary widely depending on genotype and residual enzyme activity. This suggests that individual factors and potentially compensatory mechanisms play a role in developmental outcomes. In our case, the observed acceleration in development may be related not only to standard multidisciplinary interventions but also to novel approaches, such as early tDCS and Shantala massage, which could have provided additional neurodevelopmental support. While these observations are preliminary, they highlight the potential benefit of incorporating innovative strategies in early SLOS management and warrant further study.

Studies also show developmental abnormalities of the central nervous system, such as abnormal myelination, corpus callosum defects, cerebellar anomalies, and Dandy-Walker malformation [26].

Reports of light sensitivity and sensory hyperreactivity point to the necessity of early implementation of therapy focused on sensorimotor integration [27,28,29].

Early initiation of therapeutic interventions is a response to the numerous health problems characteristic of SLOS, regardless of their severity. The flow of information between physicians and therapists has a significant impact on patient care, as striving for optimal functioning is based not only on medical and therapeutic interventions but also on education. Educating individuals in the patient’s immediate environment should therefore be one of the first supportive measures. Physiotherapy is an essential component of the treatment process due to the many motor-related issues, including muscle tone disorders, hypotonia, gross motor dysfunctions, delayed acquisition of motor skills, fine motor impairments, and the need for medical equipment and positioning devices [30,31,32,33]. Conducting research on a larger group of patients with SLOS and introducing appropriate therapeutic care standards would allow for evaluating the effectiveness of interventions. However, due to the rarity of the condition and the frequent delay in diagnosis, assembling a sufficient research group of children in the early years of life remains a challenge for initiating these interventions in a timely manner. Nonetheless, this case report aims to highlight the necessity of maintaining a holistic medical and therapeutic approach in the care of patients with SLOS.

## 5. Conclusions

### 5.1. Scientific Interpretation

This case highlights the metabolic and developmental complexity of SLOS. Despite targeted supplementation with cholesterol and cholic acid, precursor accumulation persists, likely due to residual enzyme activity of DHCR7 variants. The biochemical signature—low cholesterol with elevated 7-DHC/8-DHC—correlates with clinical severity and neurodevelopmental impairment. Although this case report presents established clinical and biochemical features of SLOS, it provides valuable longitudinal data on psychomotor development and illustrates the implementation of an individualized, multimodal, and interdisciplinary therapeutic program. The inclusion of innovative interventions, such as early tDCS and Shantala massage, offers practical insights into therapy and developmental outcomes that are not widely documented in the literature. These observations may inform clinicians managing children with SLOS and guide future research on early therapeutic strategies.

### 5.2. Importantly

HDL deficiency reflects altered lipoprotein metabolism and impaired cholesterol efflux. Persistent 7-DHC accumulation supports evidence that toxic oxysterols contribute to neurodevelopmental deficits. Therapeutic gains in psychomotor and cognitive domains suggest benefit from early, intensive multimodal intervention, though limitations remain due to the metabolic bottleneck. This underscores the need for novel therapies targeting sterol precursor reduction, such as statins, antioxidants, or gene therapy approaches currently under investigation.

### 5.3. Limitations of the Study

This study has several limitations. First, as a single-case report, the influence of individual differences on the observed outcomes cannot be ruled out, whether minor myocardial abnormalities in this patient affected developmental progress. Second, the conclusions drawn from this case cannot be generalized to all children with SLOS, as patients with different DHCR7 variants may respond differently to interventions. Third, other potential confounding factors, such as the impact of family care quality on development, were not considered. Finally, the rarity of SLOS and the delayed diagnosis in this case further limit the generalizability of the findings. Despite these limitations, we believe this case provides valuable insights for future research and clinical discussions regarding SLOS management.

## Figures and Tables

**Table 1:** Total cholesterol and sterol precursor concentrations in plasma and blood samples.

Sampling Date	Total Cholesterol	7-DHC	8-DHC
23 March 2021	17.4 mg/dL (plasma)	12.089 mg%	13.650 mg%
18 August 2021	48.9 mg/dL (plasma)	18.546 mg%	21.086 mg%
26 August 2021	77.88 mg/dL (blood)	–	–
27 September 2021	65.8 mg/dL (plasma)	18.812 mg%	21.123 mg%
15 December 2021	96 mg/dL (blood)	–	–
26 January 2022	94 mg/dL (blood)	–	–
9 May 2022	80.7 mg/dL (plasma); 111 mg/dL (blood)	20.112 mg%	24.983 mg%
28 October 2023	90 mg/dL (blood)	–	–
31 May 2024	103 mg/dL (blood)	–	–
26 March 2025	95.2 mg/dL (blood)	–	–

**Table 2:** Serum lipid fractions, vitamin D_3_, and triglycerides in selected assessments.

Sampling Date	HDL (mg/dL)	Non-HDL (mg/dL)	LDL (mg/dL)	Vitamin D_3_ (ng/mL)	Triglycerides (mg/dL)
15 December 2021	31.3	64.6	43.6	51.9	105
26 January 2022	27.8	83.2	67.98	101	76
28 October 2023	25.0	65.0	50.0	70.2	75
31 May 2024	34.1	68.9	53.58	99	77

## Data Availability

The data presented in this study is available on request from the corresponding author.

## Abbreviations

AAC	Augmentative and Alternative Communication
7-DHC	7-dehydrocholesterol
8-DHC	8-dehydrocholesterol
SLOS	Smith-Lemli-Opitz Syndrome
WES	Whole Exome Sequencing
PEG	Percutaneous Endoscopic Gastrostomy
tDCS	Transcranial Direct Current Stimulation
